# Supplementary figures and images for: Genetic analysis of activin/inhibin β subunits in zebrafish development and reproduction
Source: PLoS Genet. 2022 Dec 5;18(12):e1010523. doi: 10.1371/journal.pgen.1010523 (PMC9754609; doi:10.1371/journal.pgen.1010523)

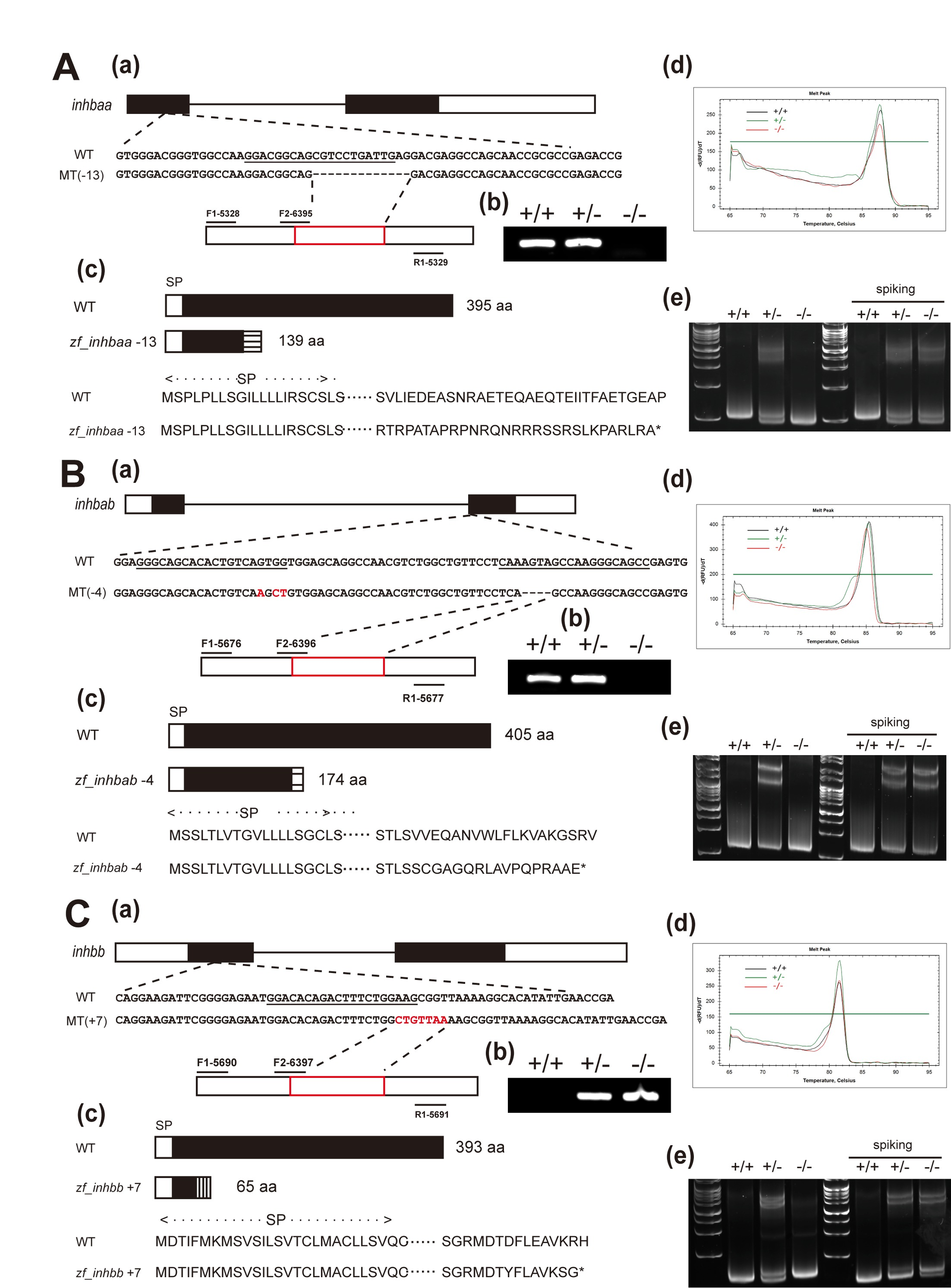

Supplement: S1 Fig — (A) Mutagenesis of inhbaa and mutant characterization. (a) Schematic illustration of the genomic structure of zebrafish inhbaa gene. The underlined sequence indicates CRISPR/Cas9 target site. The dashed line indicates the deleted sequence (-13 bp) of zebrafish inhbaa. (b) The expression of mutated transcript in the ovary. RT-PCR was performed on total RNA extracted from the ovary with a mutant-specific primer (F2-6395) overlapping with the deleted sequence. (c) Schematic representation of inhbaa amino acid sequence. The mutation is expected to introduce a premature stop codon (*). (d) Genotyping by HRMA with the primer pairs of F1-5328 and R1-5329; (e) HMA confirmation of different genotypes of inhbaa mutant. (B) Mutagenesis of inhbab and mutant characterization. (a) Schematic illustration of the genomic structure of zebrafish inhbab gene. The dashed line indicates the deleted sequence (-4 bp). (b) RT-PCR confirmation of mutation at the transcript level with a mutant-specific primer (F2-6396). (c) Schematic representation of inhbab amino acid sequence. (d) Genotyping by HRMA with the primer pairs of F1-5676 and R1-5677; (e) HMA confirmation of different genotypes of inhbaa mutant. (C) Mutagenesis of inhbb and mutant characterization. (a) Schematic illustration of the genomic structure of zebrafish inhbb gene. The inserted nucleotides are marked in red (+7 bp). (b) RT-PCR confirmation of mutation at the transcript level with a mutant-specific primer (F2-6397). (c) Schematic representation of inhbb amino acid sequence. (d) Genotyping by HRMA with the primer pairs of F1-5690 and R1-5691; (e) HMA confirmation of different genotypes of inhbb mutant. Since the homozygous mutant (-/-) and WT (+/-) were sometimes difficult to distinguish by HRMA, we spiked the samples with WT DNA to generate heterozygous product in mutant samples before PCR amplification. WT, wild type; MT, mutant. (TIF) [file pgen.1010523.s001.tif]

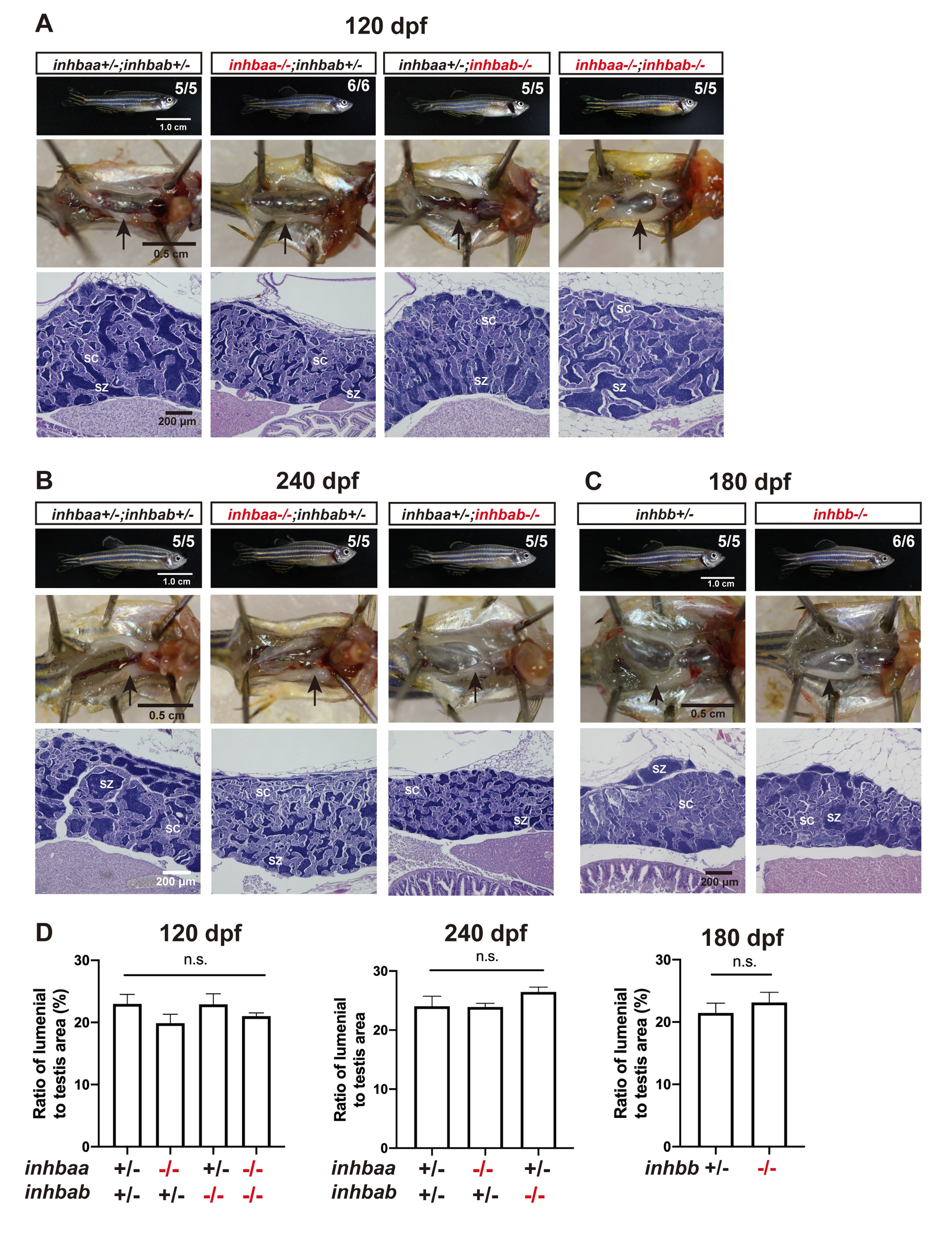

Supplement: S2 Fig — (A) Morphology, gross anatomy, and histological structure of activin βA mutant males (inhbaa-/-, inhbab-/- and inhbaa-/-;inhbab-/-) at 120 dpf. (B) Activin βA single mutant males (inhbaa-/-, inhbab-/-) at 240 dpf. (C) Activin βB mutant males (inhbb-/-) at 180 dpf. (D) Quantification of spermatozoa-filled luminal spaces in the testis at 120 and 240 dpf for activin βA mutant males and 180 dpf for βB mutant males (n = 4). (TIF) [file pgen.1010523.s002.tif]

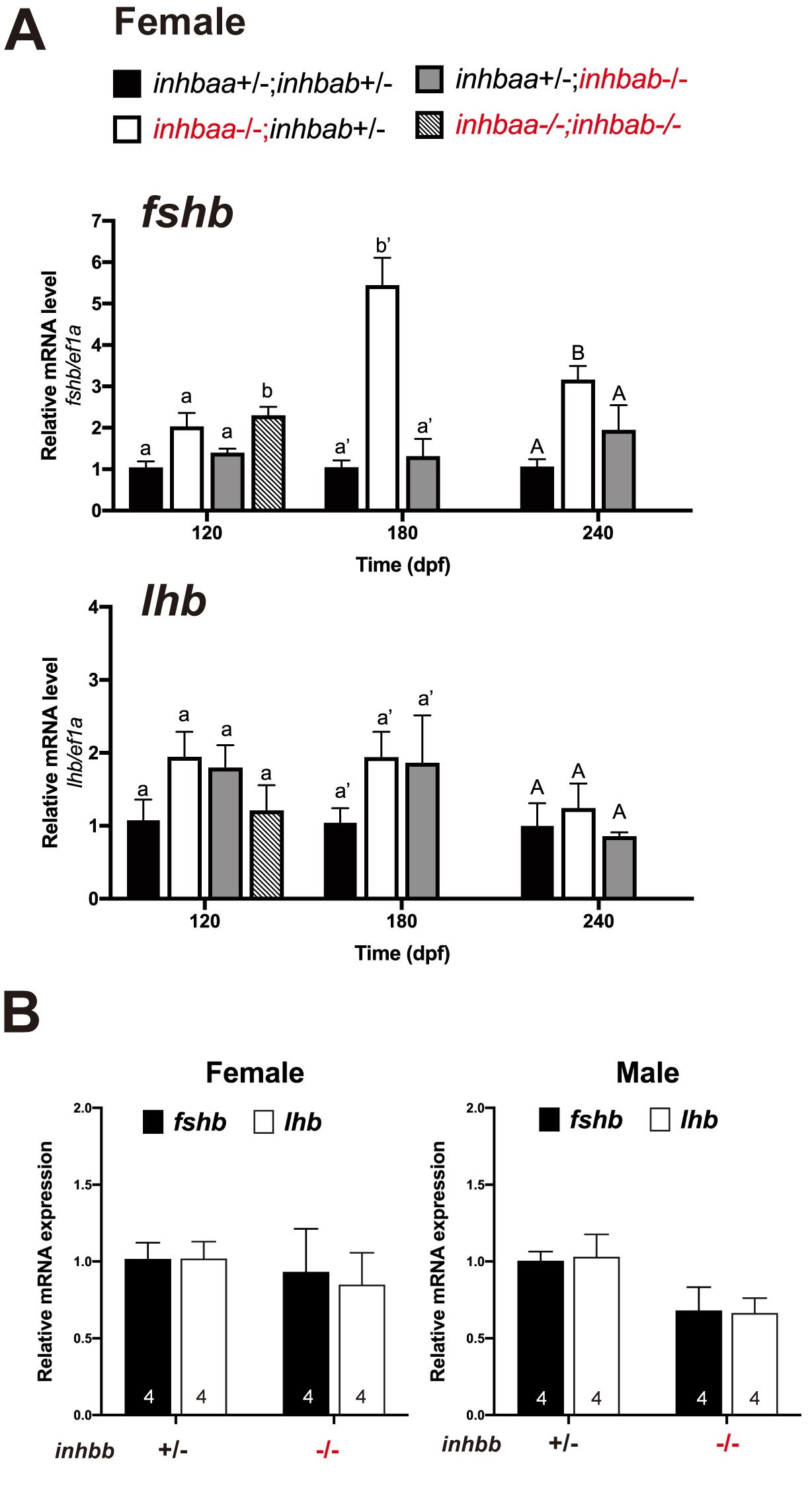

Supplement: S3 Fig — (A) Expression of fshb and lhb in the pituitary of female βA mutants at 120, 180 and 240 dpf (n = 3). The double mutant (inhbaa-/-;inhbab-/-) was not included for 180 and 240 dpf due to high mortality. Different letters in each dataset indicate statistical significance (p < 0.05). (B) Expression of fshb and lhb in the pituitary of male and female βA (inhbb) mutant at 180 dpf. (TIF) [file pgen.1010523.s003.tif]

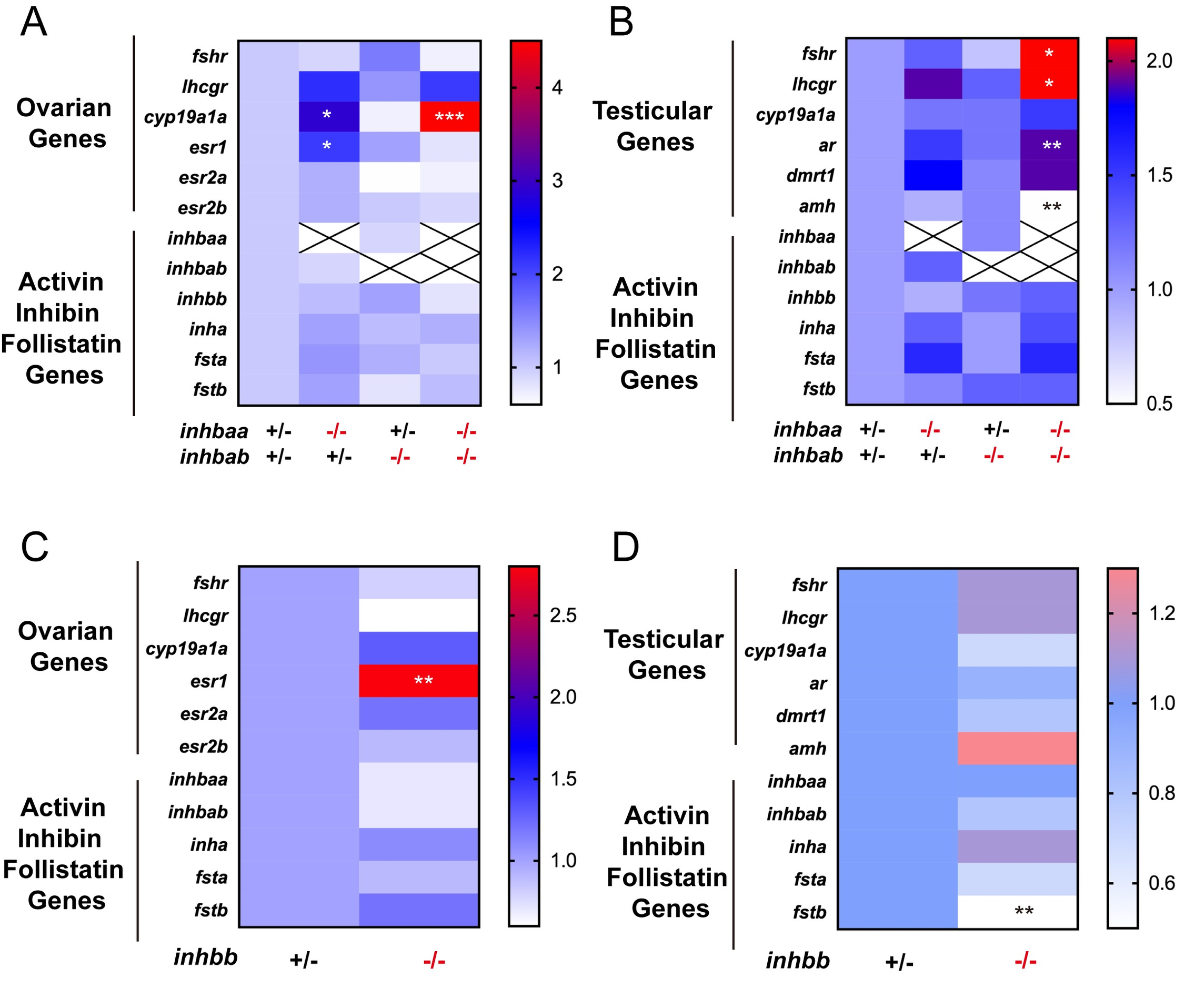

Supplement: S4 Fig — (A) Gene expression in the ovary of activin βA (inhbaa, inhbab) mutants at 90 dpf (n = 4–5). (B) Gene expression in the testis of the βA mutants at 90 dpf (n = 5). (C) Gene expression in the ovary of βB mutant (inhbb) at 180 dpf (n = 4). (D) Gene expression in the testis of the βB mutant at 180 dpf (n = 4). The expression levels are normalized to ef1a and presented as the fold change compared with the control fish. The asterisks indicate statistical significance (*p < 0.05, **p < 0.01 and ***p < 0.001). (TIF) [file pgen.1010523.s004.tif]
