# Supplementary material for: Genetic analysis of activin/inhibin β subunits in zebrafish development and reproduction
Source: PLoS Genet. 2022 Dec 5;18(12):e1010523. doi: 10.1371/journal.pgen.1010523 (PMC9754609; doi:10.1371/journal.pgen.1010523)
Supplement: S1 Table — (DOCX) [file pgen.1010523.s005.docx]

S1 Table. Primer used for CRISPR, HRMA and RT-PCR

| Gene | Primer ID | Primer Sequence | Application |
| --- | --- | --- | --- |
| *inhbaa* | 5324_*inhbaa* | TAGGACGGCAGCGTCCTGATTG | CRISPR |
|  | 5325_*inhbaa* | AAACCAATCAGGACGCTGCCGT |  |
|  | 5328_*inhbaa* | ACGCCATCCGTAAGGTACAC | HRMA |
|  | 5329_*inhbaa* | CTCCGTCTGTTCTGCCTGTT |  |
|  | 6395_*inhbaa* | GACGGCAGCGTCCTGATTGA | RT-PCR |
|  | 931_*inhbaa* | AACAGGCAGAACAGACGGAGATC |  |
|  | 932_*inhbaa* | GCAGCCGAATGTTGACGTTAGC |  |
| *inhbab* | 5672_*inhbab* | TAGGGCAGCACACTGTCAGTGG | CRISPR |
|  | 5673_*inhbab* | AAACCCACTGACAGTGTGCTGC |  |
|  | 5674_*inhbab* | TAGGCTGCCCTTGGCTACTTTG | CRISPR |
|  | 5675_*inhbab* | AAACCAAAGTAGCCAAGGGCAG |  |
|  | 5676_*inhbab* | GAGATGCCCCTGATGTCC | HRMA |
|  | 5677_*inhbab* | ACGCCGTGTATCTATGGTCTTC |  |
|  | 6396_*inhbab* | ACGTCTGGCTGTTCCTCAAA | RT-PCR |
|  | 1064_*inhbab* | AGCCCTTCGAGATCATCACCTTC |  |
|  | 1066_*inhbab* | GCCTGCTCCACCACTGACAG |  |
| *inhbb* | 5451_*inhbb* | TAGGACACAGACTTTCTGGAAG | CRISPR |
|  | 5452_*inhbb* | AAACCTTCCAGAAAGTCTGTGT |  |
|  | 5690_*inhbb* | TTGTGGACTCGGGCATCA | HRMA |
|  | 5691_*inhbb* | GGCTTTGGGAATGGGATG |  |
|  | 9379_*inhbb* | ACATACTTTCTGGCTGTTAA | RT-PCR |
|  | 933_*inhbb* | TAGGGAGGACGGCAGGGTTG |  |
|  | 934_*inhbb* | TCGTTGGAGATCAGAAAGTAGAGGC |  |
| *inha* | 520_*inha* | AGCCTCCTCTGCCAGTGTTG | RT-PCR |
|  | 521_*inha* | ATGTTGATGGAAGCGATGGTCTC |  |
| *fsta* | 963_ *fsta* | TAAAGAGACGTGTGATAATGTGGACTGTG | RT-PCR |
|  | 964_ *fsta* | ATCGCATGACTTGGCCTTGATG |  |
| *fstb* | 6253_ *fstb* | GAACAAGCGAAACAAACCC | RT-PCR |
|  | 6254_ *fstb* | GACAGCGCACGTCATAGC |  |
| *fshb* | 1794_*fshb* | TTGTTCTGGCGCTGCTGTTGC | RT-PCR |
|  | 1795_*fshb* | TTCTGGGTGTGCTGTGCCAT |  |
| *lhb* | 1797_*lhb* | GGTGTCTTCTTTCTCTTCTC | RT-PCR |
|  | 1798_*lhb* | CGGGCTCTTGTAAACGGGAT |  |
| *fshr* | 957_ *fshr* | AACATGCACATAGAGAGGATTCCCAG | RT-PCR |
|  | 958_ *fshr* | GCTCAGTAAACAGCTCCAGGC |  |
| *lhcgr* | 52_ *lhcgr* | GCGCAGATTCAGGTTATCAC | RT-PCR |
|  | 203_ *lhcgr* | GACGGCCTGAAAGGAGTAAG |  |
| *cyp19a1a* | 818_ *cyp19a1a* | TGTGCGTGTCTGGATCAATGG | RT-PCR |
|  | 819_ *cyp19a1a* | AAGCCCTGGACCTGTGAGAG |  |
| *esr1* | 1220_*esr1* | GTCTCAAAGCCATCATACTCATCAATTC | RT-PCR |
|  | 1221_*esr1* | TTCATTCGGTATAAGTGCTCCATTCC |  |
| *esr2a* | 1226_*esr2a* | CGACTTCAACAGAACCATGCTACTAG | RT-PCR |
|  | 1227_*esr2a* | CTTCACACGACCACACTCCATAATG |  |
| *esr2b* | 1230_*esr2b* | CAGTCCCTCTCAGCACCTCTTTC | RT-PCR |
|  | 1231_*esr2b* | TATCCAGCCAGCAGCATTCCAG |  |
| *ar* | 1395_ *ar* | AGCAGCAGCACCACTACCA | RT-PCR |
|  | 1396_ *ar* | TTCCTTCCTGCCTCTCGTTC |  |
| *amh* | 2573_ *amh* | GGGTGTGCATGCTACAGAAGAT | RT-PCR |
|  | 2574_ *amh* | CTCAGAAATGCAAACAGTCTGTGT |  |
| *dmrt1* | 2577_ *dmrt1* | CGATGATCGACGCTGAGAA | RT-PCR |
|  | 2578_*dmrt1* | AACCTTATAGAACGACCCCT |  |
